# Carbon Nanotube/Polymer Composites for Functional Applications

**DOI:** 10.3390/polym17010119

**Published:** 2025-01-06

**Authors:** Yoon-Ji Yim, Young-Hoon Yoon, Seong-Hwang Kim, Jeong-Hoon Lee, Dong-Chul Chung, Byung-Joo Kim

**Affiliations:** 1 Busan Textile Materials Research Center, Korea Dyeing and Finishing Technology Institute, Busan 46744, Republic of Korea; yjyim@dyetec.or.kr; 2 Korea Institute of Convergence Textiles, Iksan 54588, Republic of Korea; seonghwang@kictex.re.kr (S.-H.K.); pd2497@kictex.re.kr (J.-H.L.); 3 Department of Organic Materials and Textile Engineering, Jeonbuk National University, Jeonju 54896, Republic of Korea; 4 Department of Materials Science and Chemical Engineering, Jeonju University, Jeonju 55069, Republic of Korea

**Keywords:** carbon nanotubes, polymer composites, composite preparation, composite properties

## Abstract

Carbon nanotubes (CNTs) have garnered significant interest in the field of nanotechnology owing to their unique structure and exceptional properties. These materials find applications across a diverse array of fields, including electronics, environmental science, energy, and biotechnology. CNTs serve as potent reinforcing agents in polymer composites; even minimal additions can significantly improve the mechanical, electrical, and thermal properties of polymers. With the growing demand for polymer composites across various industries, there is an anticipation for CNT/polymer composites to evolve in increasingly diverse directions. This paper reviews recent advancements in the manufacturing techniques of various CNT/polymer composites and discusses the enhancements in their mechanical, electrical, and thermal properties. Furthermore, it explores the potential applications of these composites.

## 1. Introduction

In 1991, the discovery of carbon nanotubes (CNTs) by Iijima [1] marked a transformative development in the field of composites, revealing vast potential for addressing the inherent limitations of polymers. Characterized by their exceptional strength due to high aspect ratios, along with superior electrical and thermal conductivities, CNTs are highly effective, even in small quantities, as filler materials in polymer composites. Within these composites, CNTs are dispersed throughout the matrix, significantly enhancing the mechanical, electrical, and thermal properties of the materials [2,3,4,5]. To optimize the performance of reinforcing materials such as CNTs, the dispersion within the matrix and the interfacial bonding between the polymer and the reinforcing material are crucial. Consequently, the processing of these composites involves meticulous control of these factors, including CNT surface functionalization [6,7], use of ultrasonics [8], and structural control [9,10]. Ongoing research focuses on the physical [11,12,13,14] and chemical surface treatments [15,16,17] of reinforcing materials, alongside advancements in mechanical and chemical manufacturing technologies, aiming to achieve high-performance composites.

Furthermore, the global market for composite materials is expanding, driven by demands in sectors such as aerospace [18,19], defense [20,21], wind power generation [22,23,24], transportation machinery [25], shipbuilding [26,27], construction [28], and electrical and electronic equipment [29,30,31,32,33]. The introduction of high-functionality nanomaterials such as CNTs is expected to diversify and enhance this growth. Numerous studies have verified the versatility of CNT/polymer composites across various applications [34,35,36,37]. Given that the properties and applications of CNT/polymer composites vary depending on their manufacturing methods, this review summarizes and discusses findings from extensive research on their manufacturing processes, properties, and potential applications, presenting an overview of the current state of the field. This paper specifically examines the mechanical, electrical, and thermal properties of various CNT/polymer composites and explores their related applications.

## 2. Carbon Nanotube/Polymer Composites

### 2.1. Carbon Nanotubes

**Figure 1:**
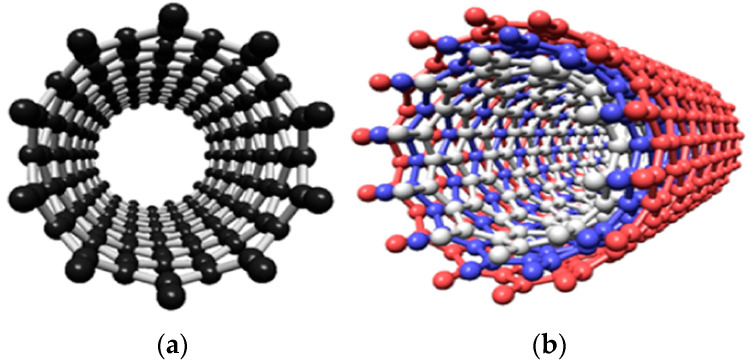
Structural comparison: (**a**) single-walled carbon nanotubes (SWCNTs) and (**b**) multi-walled carbon nanotubes (MWCNTs) [38].

CNTs are composed of rolled graphene sheets, forming tubular structures that exhibit sp^2^ hybridization of carbon atoms. Typically, CNTs are one-dimensional nanostructures with diameters ranging from 1 nm to 100 nm and can extend up to several millimeters in length. Depending on the number of concentric graphene cylinders they contain, CNTs are classified as either single-walled (SWCNTs) or multi-walled (MWCNTs), as shown in Figure 1 [38]. Because of their diameters in the nanometer range and relatively extensive lengths, CNTs have large aspect ratios and specific surface areas, displaying either semiconductor or metallic properties based on their chirality—a unique structural feature [39,40,41].

Additionally, CNTs are known for their chemical and thermal stability, alongside excellent mechanical properties. Table 1 details the key mechanical, thermal, and electrical properties of both SWCNTs and MWCNTs. The variability in their physical properties largely stems from the different structural attributes influenced by the methods and conditions used during their production [42,43].

Ongoing research explores the application of CNTs across various domains, particularly focusing on their use as reinforcing agents in functional composite materials. Even minor inclusions of CNTs can markedly enhance the mechanical strength and improve the electrical and thermal conductivities of polymers.

### 2.2. Classification of Carbon Nanotube/Polymer Composites

**Figure 2:**
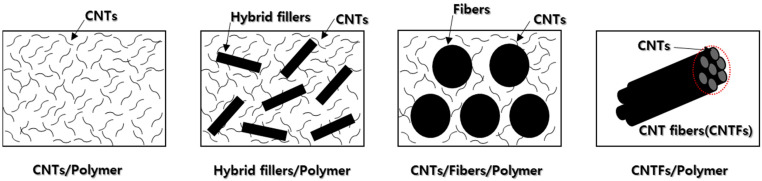
Classification of CNT/polymer composites.

When used as reinforcing agents in composite materials, CNTs enhance electrical and thermal conductivity, as well as structural integrity, owing to their exceptional strengths, moduli, and conductivities. Consequently, there is ongoing research into the production and utilization of various composite materials that incorporate CNTs. Polymer composites containing CNTs can be categorized into four distinct types, based on the composite material’s form and function (Figure 2). First, some composites use only CNTs as fillers dispersed within a polymer matrix [44,45,46,47,48,49,50,51,52,53,54,55,56,57,58,59,60,61]. Second, others combine CNTs with additional nanofillers to produce a synergistic effect within the polymer [62,63,64,65]. Third, CNTs may either be complexed on the surface of the fibers in fiber-reinforced materials or dispersed throughout the polymer matrix [66,67,68,69,70]. Lastly, CNTs are sometimes processed into macroscopic fibers and then complexed with polymers [71,72,73].

This paper primarily focuses on the first type of CNT/polymer composite, manufactured using conventional methods. The review summarizes extensive research on the manufacturing processes, properties, and applications of these composites, as reported by numerous researchers.

## 3. Preparation Methods of CNT/Polymer Composites

**Figure 3:**
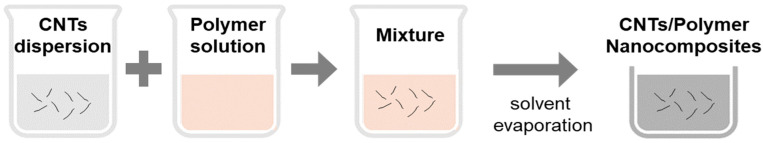
Illustration of the solution mixing method for producing CNT/polymer composites.

CNT/polymer composites can be fabricated using various techniques depending on the composite type. This section provides an overview of conventional methods for preparing CNT/polymer composites, including solution mixing, melt mixing, and in situ polymerization. Solution mixing [74,75,76], one of the simplest techniques, involves dispersing CNTs and polymers in a suitable solvent, followed by agitation using a stirrer, mixer, or ultrasonicator (Figure 3). After achieving a homogeneous dispersion, the solvent is evaporated, allowing the polymer chains to reassemble around the dispersed CNTs, forming the composite material. This method is versatile, as it is applicable to both thermoplastic and thermosetting polymers; however, it faces challenges such as high costs and solvent disposal issues.

**Figure 4:**
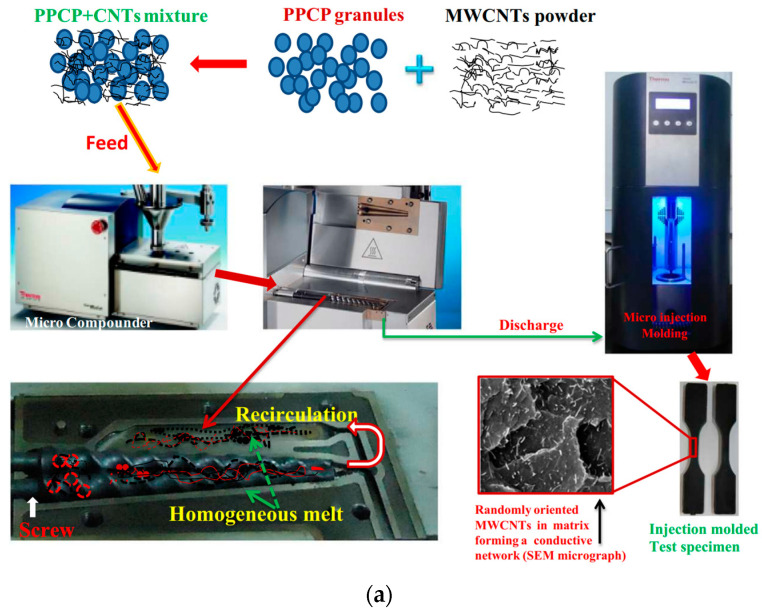

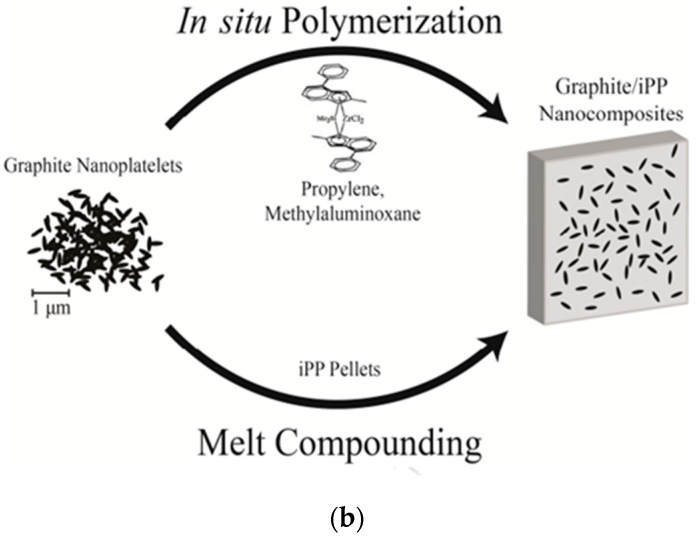
Manufacturing techniques for CNT/polymer composites: (**a**) melt-mixing processes [77] and (**b**) in situ polymerization [80].

Melt mixing [77,78,79] is another technique for preparing CNT/polymer composites, particularly well-suited for thermoplastic polymers as the matrix. This method involves using heat and mechanical shearing forces, typically through extrusion and injection molding processes (Figure 4a). In melt mixing, CNTs are uniformly dispersed within the polymer matrix because of the shear forces generated by a screw inside the machine. This method allows for effective integration of the polymer and CNTs through shearing and kneading actions. A significant advantage of melt mixing is its environmental friendliness, as it requires no solvents. However, one limitation is that achieving uniform dispersion becomes challenging at higher CNT concentrations owing to increased viscosity.

In situ polymerization [80,81,82,83] offers a different approach to preparing composites by synthesizing polymer chains directly in the presence of CNTs (Figure 4b). Initially, CNTs are dispersed uniformly in a liquid monomer or a low-molecular-weight precursor. Thereafter, an appropriate initiator is added to trigger the polymerization reaction, typically activated by heat or radiation. This process results in a composite where CNTs are intimately hybridized with the polymer molecules. In situ polymerization is favored for achieving excellent dispersion and distribution of CNTs within the composite and ensures a close interaction between CNTs and the polymer matrix. However, this method faces limitations, such as a restricted range of compatible polymers and the requirement for specialized, often costly equipment. The advantages and disadvantages of the CNT/polymer composite production method are summarized in Table 2 below.

## 4. Properties of CNT/Polymer Composites

This section reviews recent research on the mechanical, electrical, and thermal properties of CNT/polymer composites, exploring their potential applications. The discussion is structured around SWCNT/polymer and MWCNT/polymer composites, with a focus on the dispersion of CNTs, preparation methods, and the types of composites that notably influence their properties.

### 4.1. Mechanical Properties

**Figure 5:**
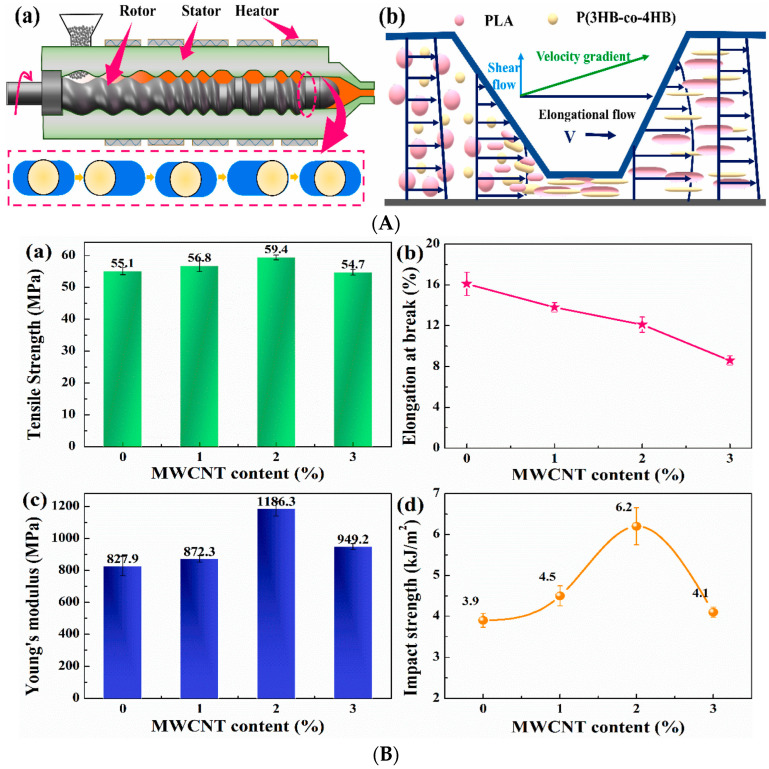
(**A**) Manufacturing method; (**a**) The ERE and rotor trajectory diagram based on elongational rheology technology, (**b**) The flow field distribution diagram of polymer melting plasticization in EFF and (**B**) mechanical properties of MWCNT/PLA/P (3HB-co-4HB); (**a**) Tensile strength (**b**) Elongation at break, (**c**) Young’s modulus (**d**) Impact strength [45].

CNTs have long been used as reinforcements for the mechanical strengthening of composites. The mechanical properties of CNT/polymer composites have been evaluated extensively, and the corresponding results are summarized in Table 3. Sun et al. [44] reported a method for improving the mechanical properties of epoxy composites by functionalizing SWCNTs via grafting with a polyamidoamine generation-0 (PAMAM-0) dendrimer, followed by adding 1 wt.% of functionalized SWCNTs to the epoxy using the solution method. The Young’s modulus, tensile strength, and K*_IC_* values of SWCNT (1 wt.%)/epoxy composites were determined to be 3.27 GPa, 74.1 GPa, and 0.58 MPa m^1/2^, respectively, and the corresponding values for PANMAN-SWCNT (1 wt.%)/epoxy composites were determined to be 3.49 GPa, 74.7 GPa, and 0.75 MPa m^1/2^, respectively. These results confirmed that the mechanical properties of the PANMAN-SWCNT (1 wt.%)/epoxy composites were better than those of the SWCNT (1 wt.%)/epoxy composite; therefore, SWCNT functionalization was effective in improving the dispersion and interfacial adhesion of the epoxy. However, the improvement in the tensile properties was not as large as expected. Li et al. [45] reported that the addition of MWCNT to PLA/P (3HB-co-4HB) blends afforded composites with special microstructures and improved strength and elastic moduli. The composites were manufactured using an extruder with different MWCNT contents, such as MWCNT/PLA/P (3HB-co-4HB). The composite with 2% MWCNT added showed the best mechanical properties: the elastic modulus, tensile strength, and impact strength of the composite were 1186.3 MPa, 59.4 GPa, and 6.2 kJ/m^2^, respectively. The mechanical properties of the composites were significantly improved compared to those of pure PLA and the PLA/P (3HB-co-4HB) blends, and it was confirmed that MWCNT contributed significantly to the increased strength of the PLA/P (3HB-co-4HB) blend (Figure 5). Liu et al. [46] fabricated PVA composites with well-dispersed functionalized SWCNTs and found that the mechanical properties of the composites with functionalized SWCNTs were significantly improved, and this improvement was attributed to the enhanced dispersion and stronger interfacial bonding with the resin achieved via proper functionalization of SWCNTs. Hayasaki et al. [47] modified SWCNTs by preparing composites using fluorinated surfactants to improve the dispersion of SWCNTs in a fluoropolymer matrix. They reported the importance of the surface properties of the SWCNTs, their compatibility with the matrix, and the role played by small amounts of dispersing aids in improving their mechanical properties. Zavala et al. [48] fabricated functionalized-SWCNT-reinforced UHMWPE composites using ultrasonic treatment and hydraulic pressing and investigated their tensile properties. The Young’s modulus and tensile strength of the f-SWCNT (0.1 wt.%)/UHMWPE composite were 1739 MPa and 51 MPa, respectively, and the tensile strength showed a 65% improvement compared to that of pure UHMWPE, which is attributed to the uniform dispersion of f-SWCNTs in the matrix. Ananthasubramanian et al. [49] functionalized SWCNTs with carboxylic acid (COOH) and silane (sily), which have hydrophilic and hydrophobic surface polarities, respectively, and prepared composites by dispersing them in a PDMS resin using a solvent-mediated dispersion process. They also reported that hydrophobic silane functionalization of SWCNTs improved their mechanical properties by improving the adhesion and dispersibility of PDMS. Mao et al. [50] synthesized MWCNTs and chlorinated polyethylene using a solution-mixing method to prepare MWCNT/CPE composites and investigated their mechanical properties. They reported that the distribution of the MWCNTs in the matrix directly affected the mechanical properties of the composites. The elastic moduli and tensile strengths of the MWCNT (10 wt.%)/CPE composites were 3.89 MPa and 21.25 MPa, respectively, meaning that the tensile strength was not significantly improved, whereas the elastic modulus was 148% higher than that of pure CPE. Díez-Pascual et al. [51] fabricated MWCNT buckypaper (BP) and composites using polyetheretherketone (PEEK) and polyphenylene sulfide (PPS) as matrices and investigated the mechanical properties of the composites through tensile and bending tests. The Young’s modulus and flexural modulus of the SWCNT-BP/PPS composite were determined to be ~3.37 MPa and 3.96 MPa, respectively, while those of the SWCNT-BP/PEEK composite were investigated to be ~4.90 MPa and 5.20 MPa, respectively. The Young’s modulus increased by ~35% and 29% compared to pure PPS and PEEK, respectively, and the flexural modulus increased by ~24% and 21% compared to pure PPS and PEEK, respectively. Therefore, the mechanical properties of the composite were significantly improved owing to the interfacial adhesion between SWCNT-BP and the matrix.

**Figure 6:**
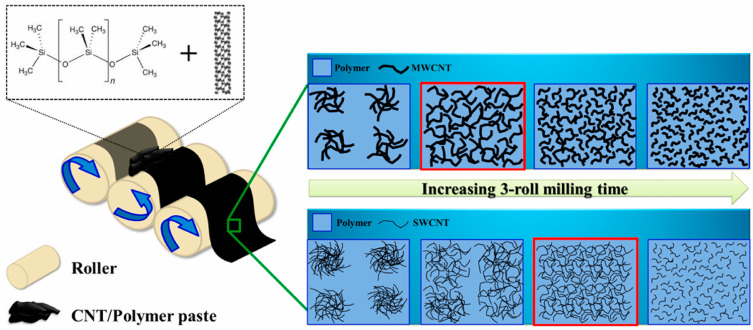
Scheme of the three-roll milling dispersion method for CNT/polymer composites according to process time: MWCNT (top) and SWCNT (bottom). The red box indicates the ideal dispersion state [52].

Hur et al. [52] dispersed SWCNTs in polymethylsiloxane (PDMS) using a three-roll mill to produce a composite. They controlled the three-roll milling conditions, viz., the milling time, milling speed (rpm), and roll spacing, to effectively disperse the SWCNTs in the matrix and measured the mechanical properties of the composites produced under each condition (Figure 6). They produced composites by adjusting the milling time between 2 and 10 min to disperse 5 wt.% SWCNTs and reported that the mechanical properties of the composites depended on the milling time. Up to 6 min, the mechanical properties improved with increasing milling time; however, when the milling time exceeded 6 min, the properties deteriorated. They concluded that the mechanical properties improved because the milling process effectively dispersed the SWCNTs. However, excessive milling deteriorated the mechanical properties. In this study, the optimal milling time was set at 6 min, and the mechanical properties of the composites were analyzed for different SWCNT contents (1–5 wt.%). The results showed that the composites became harder with increasing SWCNT content. At 5 wt.% SWCNT, the elastic modulus was 20.5 MPa, which was higher than 4.5 MPa for 1 wt.% SWCNT. Based on these results, they suggested a method to effectively disperse CNTs in the matrix using a three-roll milling method to improve the mechanical properties of the composites, thereby maximizing the utilization of the excellent properties of CNTs. The results of the studies described above have shown that the mechanical properties of composites are improved by adding CNTs, and that the dispersion and adhesion properties of CNTs in the matrix are important for further improving the mechanical properties.

### 4.2. Electrical Properties

**Figure 7:**
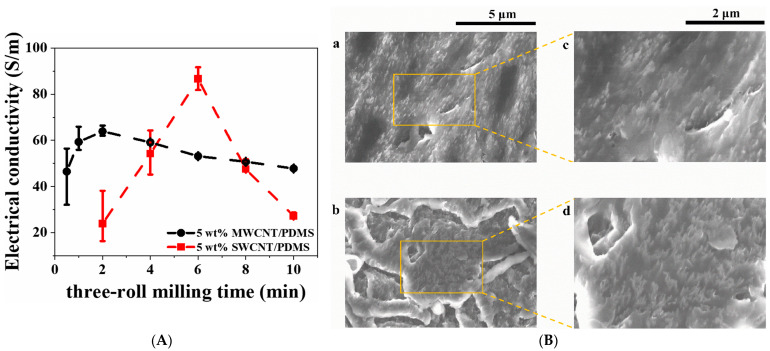
(**A**) Electrical conductivity of SWCNT and MWCNT/PDMS composites with three-roll milling process time [52] and (**B**) SEM images of the cross-sectional specimens cut from the plated-shape cavity at the center position of microinjection-molded SWCNT/PC (**a**,**c**) before annealing and (**b**,**d**) after annealing at 250 °C for 1 h [56].

Various studies have been conducted to evaluate the electrical and electromagnetic interference (EMI) shielding properties of CNT/polymer composites, with the results of some studies summarized in Table 4. Hur et al. [52] not only assessed the mechanical properties of SWCNT/PDMS composites but also studied the electrical properties of the composites produced under various three-roll milling conditions. In order to optimize the dispersion of 5 wt% SWCNTs in the matrix, the dispersion time was adjusted from 0.5 to 10 min during composite production. The composite manufactured with a milling time of 6 min exhibited the best electrical properties, with an electrical conductivity of approximately 85 S/m. The researchers also compared the electrical conductivity of SWCNT (5 wt%)/PDMS composites with that of MWCNT (5 wt%)/PDMS composites. They noted that, in the case of MWCNT (5 wt%)/PDMS composites, the composite manufactured with a milling time of 2 min showed the best electrical properties, with an electrical conductivity of approximately 65 S/m (Figure 7A). As a result of comparing the electrical conductivity of the two types of composites, the study concluded that the electrical conductivity of SWCNT/PDMS composites was lower than that of MWCNT/PDMS composites, except for the composite milled for 6 min. This was reported to be because more time was needed to evenly disperse the SWCNTs in the matrix due to their large van der Waals force. In order to optimize the electrical properties of SWCNT/PDMS and MWCNT/PDMS composites, it is essential to apply an appropriate dispersion time according to the filler’s unique physical properties. Krause et al. [53] used a dual-screw microcompounder to melt–mix SWCNTs with polypropylene to prepare SWCNT/PP composites and investigate their electrical properties. The composites were prepared by adding 0.25–1 wt% of SWCNTs. The volume resistivity of the composites was observed to decrease as the SWCNT content increased. In particular, the resistance values of the composites containing 0.075 and 0.1 wt% of SWCNTs showed a noticeable decrease, with the composite containing 0.8 wt% of SWCNTs exhibiting a volume resistivity lower than 103 Ω∙cm. In their study, the authors detected a very low electrical permeation threshold, which had not been previously reported, and suggested that the findings could be applicable not only to polypropylene but also to other thermoplastic polymer matrices. Moraiss et al. [54] investigated the effect of applying an electric field during the curing process of SWCNT/epoxy composites on their electrical conductivity. They dispersed SWCNTs in epoxy using a three-roll mill and prepared SWCNT/epoxy solutions by adding 0.1 wt% of SWCNTs. The final composites were fabricated by subjecting them to electric fields of varying strengths and frequencies during the curing process, and the electrical resistivity of the composites under different electric field application conditions was examined. The study found that the electrical resistivity of the SWCNT/epoxy composites decreased by up to 10 times with increasing electric field strength and frequency, verifying the effect of electric field application. The authors attributed the improvement in the electrical properties of the composites to the orientation and assembly of the conductive network of SWCNTs. They interpreted their experimental results using a mechanistic model based on dielectrophoresis to explain the effect of electric field application during the curing process on the electrical properties of the composites, which showed very good agreement with the experimental results. Liang et al. [55] reported their use of acid-treated SWCNTs (a-SWCNTs) as reinforcing materials; instead of polystyrene sulfonate (PSS), a representative ionic electrolyte, they used a new anionic material, tosylate (Tos), to dope poly(3,4-ethylenedioxythiophene) (PEDOT) and produced a composite material with PEDOP-Tos as the matrix. This led to the development of a flexible film with ultrahigh conductivity. The researchers stirred SWCNTs in a mixed acid solution of sulfuric acid and nitric acid, washed them with distilled water, and dried them to obtain a-SWCNTs. They explained that the acid treatment effectively improved the conductivity and dispersibility of the CNTs by removing the external defect layer on the SWCNT surface and increasing the carrier concentration through p-type doping. The electrical conductivities of pure PEDOT-Tos and a-SWCNT (35 wt%)/PEDOT-Tos composites were measured to be 38.0 ± 2.6 S/cm and 4731.6 ± 42.3 S/cm, respectively, representing an approximately 108-fold increase compared to pure PEDOT-Tos. The authors attributed this dramatic increase in electrical conductivity of the PEDOT-Tos composites with the a-SWCNT content to the following three factors: (1) high electrical conductivity due to the high aspect ratio of a-SWCNTs and effective acid treatment; (2) enhanced carrier transport due to the unique layered structure formed between PEDOT-Tos polymer chains and a-SWCNTs; and (3) strong interfacial interactions and additional charge transfer pathways between PEDOT-Tos polymer chains and a-SWCNT nanosheets, resulting in enhanced carrier transport within the composites. Thi et al. [56] used two types of CNTs (SWCNTs and MWCNTs) as reinforcements and polycarbonate (PC) as a matrix to manufacture composites through injection molding with the use of a compounder. The composites were then annealed as a post-processing treatment. The authors investigated the changes in electrical conductivity and EMI shielding properties of the composites before and after annealing. CNT contents of 1 wt% and 3 wt% were mixed with the composites, and the compounder was operated at a rotation speed of 100 rpm and a heating temperature of 260 °C. After performing injection molding on the mixed material, heat treatment was performed within a temperature range from 180 to 300 °C for a specific duration. Before annealing, the electrical conductivity values of SWCNT (1 wt%)/PC and MWCNT (1 wt%)/PC were 1.59 × 10^−14^ S/cm and 3.98 × 10^−15^ S/cm, respectively, falling within the insulating range. However, following annealing at temperatures of 230 °C and 250 °C for 2 min, the electrical conductivity increased by approximately 12 and 8 times, respectively. The authors attributed this change in the electrical properties of the composites to the rearrangement of CNTs within the composites during annealing, which resulted in the formation of a new electrical network (Figure 7B). The electrical conductivity of SWCNT (1 wt%)/PC remained between 10^−2^ S/cm and 10^−1^ S/cm after annealing, while the conductivity lines of MWCNT (1 wt%)/PC were observed to be maintained between 10^−3^ S/cm and 10^−4^ S/cm after annealing. Under the same annealing conditions, the SWCNT/PC composites showed a faster increase in electrical conductivity compared to MWCNT/PC, with a higher conductivity equilibrium. Compared to a previous study [84], the authors reported that the conductivity of the CNT (1 wt%)/PC composite after annealing was the same as that of the composite with a CNT content of 5 wt% or more before annealing. This suggests that the annealing process effectively improved conductivity. Before annealing, the composites displayed low conductivity and lacked electromagnetic shielding properties. However, following the annealing, a significant improvement was observed in both properties. The EMI shielding properties of SWCNT/PC composites with higher electrical conductivity were notably superior to those of MWCNT/PC. In particular, SWCNT (3 wt%)/PC exhibited the highest electrical conductivity and EMI shielding properties of approximately 46–51 dB, while MWCNT (3 wt%)/PC showed shielding properties in the range of 20–23 dB. The authors suggested that these newly developed composites could serve as effective EMI shielding materials in 5G technology applications.

Kathalingam et al. [57] prepared flexible composite films using SWCNTs as reinforcement and polymethyl methacrylate (PMMA) as a matrix through a simple solution method. They investigated the electrical properties of the composite films and emphasized the significance of solvent selection in uniformly dispersing SWCNTs in the PMMA polymer. In their study, they used a water–ethanol mixed solution. The analytical results showed that the electrical conductivity of the prepared SWCNT/PMMA composite film was 10.9 S/cm, which was significantly higher than that of pure PMMA. This observation suggested a strong interfacial interaction between SWCNTs and PMMA. Hussain et al. [58] fabricated composites using MWCNTs and silver-decorated MWCNTs (Ag-MWCNTs) as reinforcements, along with polyaniline (PANI) as a matrix through in situ polymerization. Various filler weight percentages ranging from 0.2 to 5% were investigated, and the electrical properties of the composites were studied. The electrical conductivity of the MWCNT/PANI composites increased with the increasing MWCNT content up to 4% but decreased when the MWCNT content reached 5%. The highest electrical conductivity was noted in the MWCNT (4%)/PANI composite, reaching 5.7 S/cm. In the case of the Ag-MWCNT/PANI composite, the maximum electrical conductivity was observed at a composition of 2%, which measured 11.894 S/cm. It was explained that when the content of Ag-MWCNTs exceeds 2%, the Ag-MWCNTs aggregate within the composite, leading to a reduction in the conductivity of the composite. In addition, the electrical properties of the composite with Ag-MWCNTs incorporated into it were found to be significantly higher than those of MWCNTs, as Ag has a higher conductivity compared to pure MWCNTs. Because both materials exhibit excellent electrical conductivity, the high electrical conductivity value of the Ag-MWCNT/PANI composite is likely the result of a synergistic effect between the two materials. The research results indicate that Ag-MWCNTs are a more effective filler for increasing the electrical conductivity of composites compared to MWCNTs, and that this improvement is attributed to the enhanced interaction between Ag-MWCNTs and PANI. Avadhanam et al. [59] fabricated SWCNT-PANI/PU composites through a two-step process and evaluated their electrical conductivity and EMI shielding properties. They first fabricated a SWCNT-PANI core–shell as a filler for the composites via in situ polymerization. Subsequently, they incorporated 0.1–5 wt% of SWCNT-PANI, dispersed it within the PU polymer using ultrasonication, and cured it to fabricate the final composites. Analysis of the electrical conductivity revealed that pure PU exhibited a value of 5.26 × 10^−13^ S/cm, while the SWCNT-PANI (0.1 wt%)/PU composite exhibited a conductivity of 4.83 × 10^−8^ S/cm. The electrical conductivity of the 0.1 wt% composite was significantly increased compared to that of pure PU, indicating the formation of a conductive network due to the filler. Furthermore, the maximum electrical conductivity of 6.28 × 10^−4^ S/cm was achieved by the SWCNT-PANI (3 wt%)/PU composite, with the electrical conductivity decreasing with further increases in the filler content. This behavior was explained to be a result of agglomeration at higher SWCNT-PANI loadings. To evaluate the EMI shielding properties of the SWCNT-PANI/PU composite, it was applied to a GFRP substrate, and the EMI shielding properties of the SWCNT-PANI (3 wt%)/PU composite in the 11–12 GHz range were determined to be 11 dB. Similar to the electrical conductivity results, the EMI shielding properties decreased with filler loadings exceeding 3 wt% due to agglomeration. The findings from the abovementioned studies have shown that the addition of CNTs improves the electrical and EMI shielding properties of composites, and the dispersion of CNTs within the matrix and the surface properties of CNTs are important to consider for further improvements.

### 4.3. Thermal Properties

Many researchers have attempted to improve the thermal properties of CNT/polymer composites by functionalizing CNTs and employing various dispersion and fabrication techniques. The results of some of these studies are summarized in Table 5.

**Figure 8:**
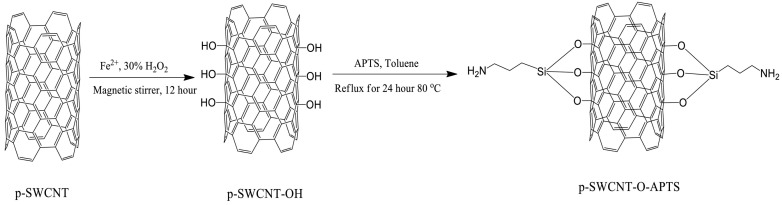
Synthesis reaction of p-SWCNT-OH and p-SWCNT-O-APTS [60].

For example, Yanmaz et al. [60] modified SWCNTs through hydroxylation and silane reactions, subsequently dispersing them in PVA resin using a solution-based method (Figure 8). Their use of sodium dodecyl sulfate (SDS) enabled the uniform dispersion of SWCNTs within the matrix. They analyzed the thermal properties of the composites under different manufacturing conditions, including filler content, surface modification method, and surfactant addition. The glass transition temperature of the composites was found to increase with increasing filler amount. The Tg of PVA was 72.8 °C, while the composites with added SWCNTs and modified SWCNTs exhibited increases of 0.5–3.4 °C compared to pure PVA, and those with added SDS showed increases of 3.8–5.9 °C. The highest Tg value was observed in the p-SWCNT-OH (1 wt%) PVA/SDS composite, which was approximately 92.4 °C. The authors attributed this improvement to the effective dispersion of SWCNTs within the matrix, promoted by surface modification and the use of SDS, which prevented agglomeration and strengthened the interaction between SWCNTs and the matrix. In addition, they reported that the thermal properties of PVA, such as its glass transition temperature, are also affected by surface modification, the filling ratio of SWCNTs, and the presence of surfactants. Similarly, Zanjanijam et al. [61] investigated the thermal properties of SWCNT/poly(vinyl chloride) (PVC) composites prepared with varying SWCNT contents. The inclusion of SWCNTs improved the thermal properties of the composites, thereby reducing the weight loss during the degradation process of PVC. At 600 °C, the residual mass of PVC was 0.19%, but the addition of 1% SWCNTs significantly increased the residual mass to 59.5%, indicating that the thermal stability of the composites could be effectively improved. Hussain et al. [58] also investigated the thermal properties of MWCNT/PANI and Ag-MWCNT/PANI composites. Their thermogravimetric analysis revealed that the thermal stability of the composites was superior to that of PANI alone, attributed to the ability of MWCNTs to delay the thermal decomposition of the PANI composite. The weight loss of the Ag-MWCNT (0.6%)/PANI composite was approximately 55% at around 600 °C, with Ag-MWCNTs demonstrating greater thermal stability compared to MWCNTs. Consequently, the Ag-MWCNT/PANI composite exhibited 12–15% lower weight loss than the MWCNT/PANI composite, demonstrating its superior thermal stability. Overall, the studies listed above confirm that the addition of CNTs improves the thermal properties of composites, primarily due to the excellent thermal properties of CNTs. Furthermore, the dispersion of CNTs within the matrix and their surface properties are essential factors contributing to the enhancement of the composites’ thermal properties.

## 5. Functional Applications

### 5.1. Aerospace and Automobiles

CNT/polymer composites are being utilized in various industrial fields due to their excellent mechanical, electrical, and thermal properties, as has been previously confirmed. In particular, in the aerospace and automobile industries, there is a growing demand for high-performance, lightweight materials to enhance fuel efficiency and comply with environmental regulations. As a result, functional composites such as CNT/polymer composites are emerging as promising alternatives to traditional metal materials, leading to an acceleration of research in this area [85,86,87]. The mechanical and thermal properties of composites are crucial for their application in these industries, given the thermal cycle environments created by drastic temperature variations in space, during aircraft takeoffs and landings, and in vehicle operation. Anvari et al. [88] emphasized the importance of confirming changes in the mechanical properties of composites when subjected to thermal cycles for application in these fields.

### 5.2. Sensors

Recently, structural health monitoring (SHM) technology, which involves the integration of sensors into structures to monitor their health, has gained attention as a critical technology in industries such as aerospace, automobiles, construction, and civil engineering, where structural safety is paramount. Research is also being conducted to develop CNT-based self-sensing SHM composites that can function as built-in sensors for detecting damage to CNT/polymer composites by leveraging the electrical properties of CNTs [89,90,91]. Shirodkar et al. [91] reported on the damage detection characteristics by evaluating and analyzing the change in resistance to deformation of MWCNT/polymer composites, and they suggested a correlation between mechanical and electrical behaviors. This is based on the principle that when a crack occurs in a composite, the electrical connection is partially broken, which increases the resistance. They stated that MWCNT can be applied as a sensor capable of real-time deformation and damage evaluation of composites. CNT/polymer composites are not only suitable for structural sensors but also for applications in wearable technology for health monitoring, as well as in the biomedical field [92,93,94]. Choi et al. [92] reported the fabrication of CNT/polymer composite fibers with piezoresistive properties and their applicability as fiber sensors for wearable devices. They explained that the developed composite fibers have high elasticity and controllable electrical properties, making them suitable for the development of various wearable devices as stretchable strain sensors.

### 5.3. EMI Shielding

**Figure 9:**
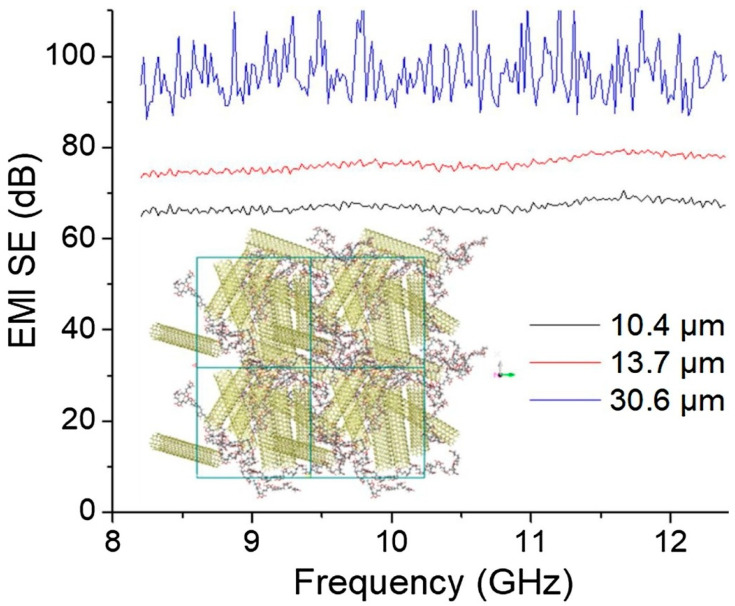
Simulation model containing CNT/polymer and EMI SE of the composites [97].

In the context of advancing technologies such as 5G communication, electric vehicles, and autonomous driving, the miniaturization and increased integration of electronic components have led to challenges related to electromagnetic interference and overheating. CNT/polymer composites, with their excellent electrical conductivity, EMI shielding properties, and exceptional thermal stability, are recognized as materials capable of addressing these issues [95,96,97,98]. Hayashida et al. [96] investigated the EMI shielding properties of CNT/polymer composites and showed shielding performance over a wide frequency band up to 110 GHz, suggesting the possibility of solving EMI problems in advanced electronic and communication devices. Mo et al. [97] fabricated a CNT/polymer composite film and confirmed its applicability as an EMI shielding material. They improved the electrical conductivity of the composite film through a doping process and developed a material with excellent shielding properties of 96.3 dB at a thickness of 0.031 mm. They explained that the composite film they developed is suitable as an EMI shielding material for electronic devices that are becoming smaller and smarter (Figure 9).

### 5.4. Energy Harvesting

**Figure 10:**
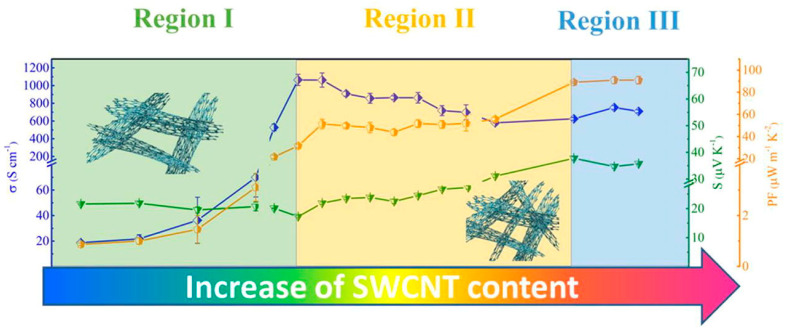
Room-temperature electrical conductivities, Seebeck coefficients, and power factors as a function of CNT loading composites, with schematic illustration of CNT/polymer composites [99].

Moreover, these composites exhibit thermoelectric properties that can convert thermal energy into electrical energy and vice versa, making them attractive for applications in the energy sector. These thermoelectric materials can be used to develop technologies that contribute to energy savings and are being used in various industries, such as automobile, aerospace, semiconductor, biology, and electronics [99,100,101]. Wei et al. [99] reported the results of a study on the thermoelectric properties of CNT/polymer composite films fabricated. The composite films showed different temperature dependence of thermoelectric performance depending on the CNT content, and they showed very stable thermoelectric performance even after 1000 bending cycles. They suggested the possibility of applying CNT/polymer composite films to energy harvesting technology (Figure 10).

### 5.5. Biomedical Engineering

**Figure 11:**
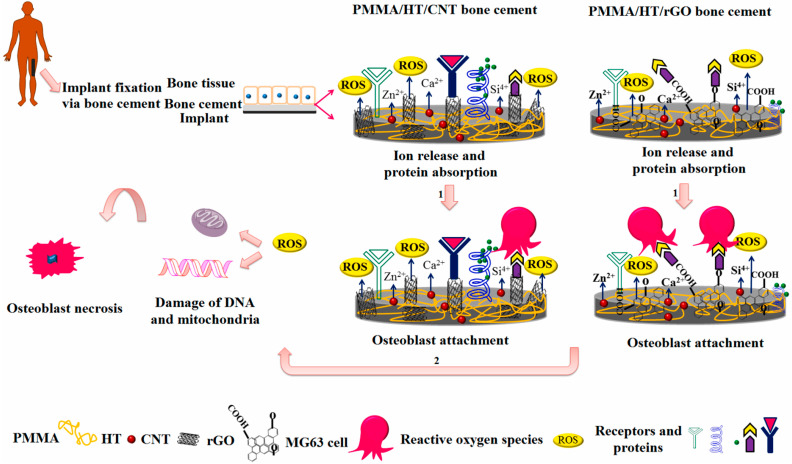
Schematic illustration of PMMA-based bone cement for CNT and cell attachment; positive (pathway 1) and negative effect (pathway 2) [102].

In addition, research is actively being conducted to explore the use of CNT/polymer composites in artificial joints and implants, given their excellent mechanical properties and biocompatibility. The potential for these materials in biomedical applications holds promise for improving human lives [102,103,104,105]. Pahlevanzadeh et al. [102] fabricated a CNT/polymer-based bone cement, evaluated its mechanical and biological properties, and investigated its applicability as a biomaterial for implant fixation. They explained that the fabricated composite had excellent mechanical properties and biocompatibility, so it could be applied as a bone cement material suitable for implant fixation (Figure 11).

## 6. Conclusions and Future Perspectives

In this review, we investigated the results of studies on the mechanical, electrical, and thermal properties of CNT/polymer composites, as reported in the literature, and we described their applicability in various industrial fields. Specifically, we reviewed and summarized the recent developments related to the effects of using CNTs as reinforcement in polymer composites on their mechanical, electrical, and thermal properties. In summary, the excellent properties of CNT/polymer composites stem from the presence of CNTs, with the dispersion state of CNTs in the matrix playing a crucial role. The properties of these composites are affected by both the inherent properties and the dispersion state of the CNTs, with any agglomeration of CNTs having a negative effect on the physical properties. It was confirmed that CNT/polymer composites have the potential to be utilized across a wide range of industries, including the aerospace, automotive, construction, sensor, and medical industries. However, among the various types, polymer composites containing only CNTs have limited CNT content that can be added, and their mechanical properties are inferior to those of conventional fiber-reinforced composites (FRPs) despite their superior electrical and thermal properties. To overcome these limitations, research on manufacturing hybrid composites by adding fillers such as CNTs to FRP composites is gradually increasing. In the case of these CNT hybrid composites, synergistic effects of various materials can be obtained, but since several materials are composited, they have complex molding conditions, and due to various types of materials, interfacial property control is particularly more important. Therefore, research on optimizing the manufacturing method of CNT hybrid composites is essential, and it is thought that practical application and expansion of the use of CNT composites in various fields will be possible through such continuous and creative research.

## Figures and Tables

**Table 1:** Comparative physical properties of SWCNTs and MWCNTs [42,43].

Properties	SWCNTs	MWCNTs
Diameter (nm)	1–2	10–100
Tensile strength (GPa)	50–500	10–60
Tensile stiffness (TPa)	~1.4	~1
Thermal conductivity (WmK^−1^)	~6000	~3000
Specific resistance (μΩcm)	5–50	5–50

**Table 2:** Advantages and disadvantages of CNT/polymer composite production methods.

**Preparation Methods**	**Advantages**	**Disadvantages**
Solution mixing method	- No restrictions on materials	- High costs - Solvent disposal
Melt-mixing processes	- Environmentally friendly method	- Cannot manufacture high-concentration CNT/polymer composites
In situ polymerization	- Excellent dispersion and distribution of CNTs	- Limitations on compatible polymers - Requires special and expensive equipment

**Table 3:** Mechanical properties of CNT/polymer composites.

CNT/Polymer	Dispersion	Preparation Method	Mechanical Properties	Ref.
SWCNT (1 wt.%)/Epoxy	-	Solution method	Young’s modulus: 3.27 GPa Tensile strength: 74.1 GPa *K*_IC_: 0.58 MPa m^1/2^	[44]
PAMAM-SWCNT (1 wt.%)/ Epoxy	Polyamidoamine generation-0 (PAMAM-0) dendrimer	Solution method	Young’s modulus: 3.49 GPa Tensile strength: 74.7 GPa *K*_IC_: 0.75 MPa m^1/2^	[44]
MWCNT(2%)/PLA/ P (3HB-co-4HB)	-	Melting method	Young’s modulus: 1186.3 MPa Tensile strength: 59.4 GPa Impact strength: 6.2 kJ/m^2^	[45]
f-SWCNT(0.8 wt.%)/PVA	Multiple surface hydroxyl groups	Solution method	Tensile modulus: 4.3 GPa Tensile yield strength: 107 MPa	[46]
Fluorocarbon-SWCNT(0.2 wt.%)/PVDF/FDPA(0.2 wt.%)	Fluorine-modified SWCNT/ FDPA fluorocarbon-based surfactant	Melting method	Young’s modulus: 2632 MPa Tensile strength: 95 MPa	[47]
f-SWCNT(0.1 wt.%)/ UHMWPE	Functionalized SWCNTs	Hydraulic pressing method	Young’s modulus: 1739 MPa Tensile strength: 51 MPa	[48]
sily-SWCNT (1 wt.%)/PDMS	Silane-functionalized SWCNTs	Solution method	Elastic modulus: 4.15 MPa Tensile strength: 1.58 MPa	[49]
MWCNT (10 wt.%)/CPE	-	Solution method	Elastic modulus: 3.89 MPa Tensile strength: 21.25 MPa	[50]
SWCNT-BP/PPS	Buckypaper	Hot-pressing method	Young’s modulus: 3.37 MPa Flexural modulus: 3.96 MPa	[51]
SWCNT-BP/PEEK	Buckypaper	Hot-pressing method	Young’s modulus: 4.90 MPa Flexural modulus: 5.20 MPa	[51]
SWCNT (5 wt.%)/PMDS	3-roll milling method	Solution method	Elastic modulus: 20.5 MPa	[52]

**Table 4:** Electrical and electromagnetic interference (EMI) shielding properties of CNT/polymer composites.

CNT/Polymer	Dispersion	Preparation Method	Electrical Properties/ EMI Shielding Properties	Ref.
SWCNT (5 wt%)/PMDS	Three-roll milling method	Solution method	Electrical conductivity: ~85 S/m	[52]
MWCNT (5 wt%)/PMDS	Three-roll milling method	Solution method	Electrical conductivity: ~65 S/m	[52]
SWCNT (0.8 wt%)/PP	-	Melting method	Volume resistivity: ~10^2^ Ω∙cm	[53]
SWCNT (0.1 wt%)/epoxy	Three-roll milling method	Solution method	Volume resistivity: ~10^2^ Ω∙cm	[54]
Acid-SWCNT (35 wt%)/ PEDOT-Tos	Acid-functionalized SWCNTs/ ultrasonication	Solution method	Electrical conductivity: ~4731.6 S/cm	[55]
SWCNT (1 wt%)/PC	Annealing	Melting method	Electrical conductivity: ~1.59 × 10^−14^ S/cm EMI shielding: 46–51 dB	[56]
MWCNT (1 wt%)/PC	Annealing	Melting method	Electrical conductivity: ~3.98 × 10^−15^ S/cm EMI shielding: 20–23 dB	[56]
SWCNT/PMMA	Ultrasonication	Solution method	Electrical conductivity: 10.9 S/cm	[57]
AG-MWCNT (2%)/PANI	-	In situ polymerization	Electrical conductivity: 11.894 S/cm	[58]
SWCNT-PANI (3 wt%)/PU	Ultrasonication	Solution method	Electrical conductivity: ~6.28 × 10^−4^ S/cm EMI shielding: 20–23 dB	[59]

**Table 5:** Thermal properties of CNT/polymer composites.

CNT/Polymer	Dispersion	Preparation Method	Thermal Properties	Ref.
p-SWCNT-OH (1 wt%)/PVA /SDS	Functionalized SWCNTs/surfactant	Solution method	Tg values: ~92.4 °C	[60]
SWCNT (0.8 wt%)/PVC	Ultrasonication	Solution method	Residual mass (600 °C): 59.50%	[61]
Ag-MWCNT (0.6%)/PANI	-	Solution method	Weight loss (600 °C): ~55%	[58]
